# The role and regulation of erythropoietin (EPO) and its receptor in skeletal muscle: how much do we really know?

**DOI:** 10.3389/fphys.2013.00176

**Published:** 2013-07-15

**Authors:** Séverine Lamon, Aaron P. Russell

**Affiliations:** Centre for Physical Activity and Nutrition Research, School of Exercise and Nutrition Sciences, Deakin UniversityBurwood, VIC, Australia

**Keywords:** erythropoietin, erythropoietin receptor, skeletal muscle, signaling, cytokines

## Abstract

Erythropoietin (EPO) primarily activates erythroid cell proliferation and growth and is active in several types of non-hematopoietic cells via its interaction with the EPO-receptor (EPO-R). This review focuses on the role of EPO in skeletal muscle. The EPO-R is expressed in skeletal muscle cells and EPO may promote myoblast differentiation and survival via the activation of the same signaling cascades as in hematopoietic cells, such as STAT5, MAPK and Akt. Inconsistent results exist with respect to the detection of the EPO-R mRNA and protein in muscle cells, tissue and across species and the use of non-specific EPO-R antibodies contributes to this problem. Additionally, the inability to reproducibly detect an activation of the known EPO-induced signaling pathways in skeletal muscle questions the functionality of the EPO-R in muscle *in vivo*. These equivocal findings make it difficult to distinguish between a direct effect of EPO on skeletal muscle, via the activation of its receptor, and an indirect effect resulting from a better oxygen supply to the muscle. Consequently, the precise role of EPO in skeletal muscle and its regulatory mechanism/s remain to be elucidated. Further studies are required to comprehensively establish the importance of EPO and its function in skeletal muscle health.

## EPO, A pleiotropic hormone

Erythropoietin (EPO) is a cytokine hormone primarily dedicated to erythroid progenitor cell development and proliferation. After birth, EPO is essentially synthetized in the peritubular fibroblast-like cells located in the cortex of the kidneys (Maxwell et al., 1993, 1997). Its production is largely stimulated by tissue hypoxia and results in an increased number of circulating erythrocytes and, consequently, in an improved oxygen supply to the active tissues (Jelkmann, 2004; Noguchi et al., 2008; Jelkmann, 2011). In the last decade, numerous studies reported that apart from its hematopoietic effect, EPO was active locally in many other tissues, including endothelial, neural, muscle, cardiovascular and renal tissues, in response to physical or metabolic stress. Accordingly, the expression of the specific EPO receptor (EPO-R) extends beyond hematopoietic cells [previously reviewed in Noguchi et al. (2008)]. Based on these findings, pleiotropic functions have been proposed for EPO, which has notably been suggested to be involved in protection against oxidative stress in neuronal cells (Zaman et al., 1999), in neovascularization in uterine angiogenesis (Yasuda et al., 1998) and in maintenance and repair of myocardium (Tada et al., 2006). This review focuses on the role of EPO and its specific receptor (EPO-R) in skeletal muscle tissue.

## Structure and function of EPO and its receptor

The circulating human EPO protein is made of 166 amino acids (Miyake et al., 1977; Jacobs et al., 1985; Lin et al., 1985). Two antiparallel pairs of α-helical bundles linked by two disulphide bridges between Cys residues (Jacobs et al., 1985; Lin et al., 1985) form a glomerular structure analogous to the structure of growth hormone despite little similarity in amino acid sequence (Wen et al., 1994). EPO is a highly glycosylated protein. The molecular mass of the peptide backbone of EPO is 18 kDa, whereas the molecular mass of the glycoprotein is 30.4 kDa. Human EPO includes three N-linked (Asn24, Asn38, Asn83) and one O-linked (Ser126) acidic oligosaccharide side chains (Dordal et al., 1985; Sasaki et al., 1987). Structural modifications or permutations of the carbohydrate chains can alter the molecular mass, biological activity, electric charge or immunoreactivity of the EPO protein (Wide and Bengtsson, 1990; Wasley et al., 1991; Hammerling et al., 1996; Jelkmann, 2007).

EPO primarily exerts its effect by binding a cytokine class I receptor superfamily member, the EPO-receptor (EPO-R) (D'Andrea and Zon, 1990; Youssoufian et al., 1993). The EPO-R is a 508 amino acids transmembrane receptor (Noguchi et al., 1991) including an extracellular domain containing a WSXWS motif, a single transmembrane hydrophobic domain and a variable cytoplasmic domain (Youssoufian et al., 1993). The active form of the EPO-R on the surface of erythroid progenitor cells is a homodimer. Different models of hematopoietic and non-hematopoietic cell lines have allowed to establish that EPO binding induces a conformational change in two adjacent monomeric EPO-R molecules, which leads to the phosphorylation of the cytoplasmic domain-associated JAK2 proteins (Constantinescu et al., 1999; Remy et al., 1999), their activation of a JAK2 Tyr kinase (Witthuhn et al., 1993) and the consequent phosphorylation of the EPO-R (Miura et al., 1991; Dusanter-Fourt et al., 1992). EPO-induced JAK2 activation provokes STAT5 phosphorylation and translocation to the nucleus where it activates the transcription of its target genes (Damen et al., 1995; Pallard et al., 1995; Penta and Sawyer, 1995; Klingmuller et al., 1996; Quelle et al., 1996). In addition, JAK2 activation has the potential to trigger various signaling cascades in response to EPO binding. The Ras/mitogen-activated kinase (MAPK) pathway and the phosphatidylinositol 3-kinase (PI3)/Akt pathway are notably activated by EPO in interleukin-3-dependent cell lines expressing high levels of EPO-R (Damen et al., 1993; Miura et al., 1994; Constantinescu et al., 1999; Fisher, 2003).

Alternatively, it was previously reported that the EPO-R could physically associate and interact with the common β receptor (βcR) subunit (Jubinsky et al., 1997; Blake et al., 2002), suggesting a potential role of βcR in EPO signaling. However, βcR knockout mice exhibit normal hematopoiesis (Scott et al., 2000). It was therefore proposed that a heteroreceptor complex comprising both EPO-R and βcR could, at least partially, mediate the non-hematopoietic functions of EPO (Brines et al., 2004).

## Expression of the EPO-R and its role in intracellular signaling

Ogilvie and colleagues first reported that the EPO-R gene and protein were expressed in primary satellite cells isolated from mouse skeletal muscle and in cultured C_2_C_12_ myoblasts. EPO-R gene expression was downregulated during the differentiation of C_2_C_12_ myoblasts to myotubes (Ogilvie et al., 2000), a phenomenon analogous to that observed during erythropoiesis where EPO-R is not expressed in mature red blood cells. However, EPO-R gene expression increased with myoblast proliferation (Jia et al., 2009), suggesting a role in regeneration. Mouse embryos expressing the lacZ reporter gene, driven by the human EPO-R promoter, showed *in-situ* hybridization staining in the region of the visceral arches and base of limbs, suggesting a role for EPO-R in the developing muscle (Ogilvie et al., 2000). However, EPO-R mRNA expression was not detected in the skeletal muscle of adult transgenic mice expressing the human EPO-R gene (Liu et al., 1994, 1997), although it was not detected in any other non-hematopoietic tissue either. Presence of the rat and human EPO-R mRNA and protein was reported in rat L6 myoblasts and in human primary myoblast cultures, respectively (Launay et al., 2010). In contrast, EPO-R gene and protein expression was not detected in rat myoblasts isolated from normal muscle, while a transient, unexplained induction was observed 1 and 7 days following a mechanical-induced muscle injury (Rotter et al., 2008). The EPO-R protein was detected in cross sections of human skeletal muscle tissue and was primarily localized in the region of vascular cells and of the skeletal muscle membrane (sarcolemma) (Lundby et al., 2008a; Rundqvist et al., 2009). The presence of EPO-R mRNA and protein in skeletal muscle biopsies has been reported while EPO-R mRNA has been detected in isolated human muscle fibers and human satellite cells. Finally, the EPO-R protein was detected in total muscle extracts from muscle biopsies (Rundqvist et al., 2009; Christensen et al., 2012a). These results strongly suggest that the EPO-R mRNA is present in muscle tissue, although differences may exist between species and cell lines.

It was suggested that expression of the EPO-R gene was triggered by EPO stimulation. C_2_C_12_ cells cultured in the presence of EPO showed a substantial increase in EPO-R gene expression (Ogilvie et al., 2000) and an increase in EPO-R protein levels in both normoxic and hypoxic conditions (Jia et al., 2009). EPO transgenic mice with chronically elevated circulating levels of EPO displayed higher EPO-R mRNA levels in primary myoblasts when compared with wild-type mice. Inversely, knockdown of circulating EPO levels did not lead to any change in EPO-R gene expression in transgenic mouse muscle (Hagstrom et al., 2010a; Mille-Hamard et al., 2012). Similarly in human muscle, EPO-R gene expression was not modified by acute EPO administration (Lundby et al., 2008a).

Discrepancies in the measurement of EPO-R mRNA and protein levels appear to be related to potential species differences and the use of *in vitro* verses *in vivo* models. Studies using human primary muscle cells and mouse muscle *in vivo* will help to clarify some of these inconsistencies. Furthermore, concerns surrounding antibody specificity (Elliott et al., 2006; Kirkeby et al., 2007) (discussed in detail below) suggest that conclusions drawn about EPO-R protein expression, role and functionality in skeletal muscle need to be considered with caution.

**Figure 1:**
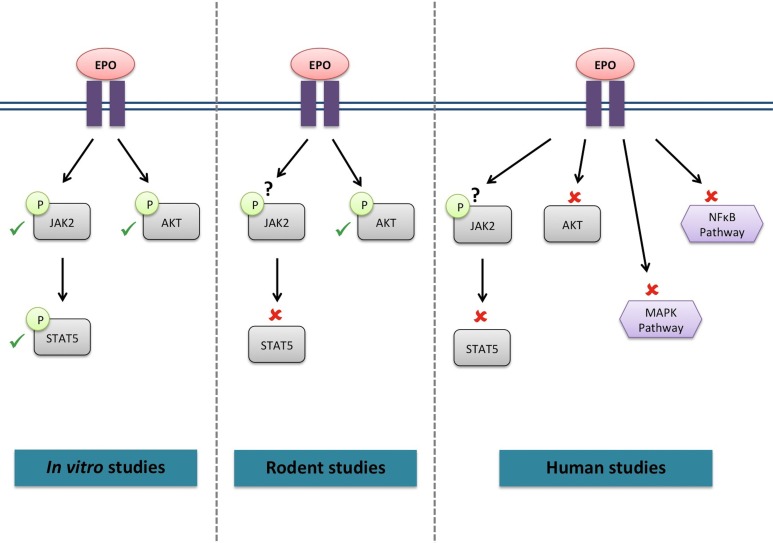
**Signaling cascades activated by EPO in skeletal muscle**. ✓, activated by EPO; ✘, not activated by EPO; ?, not investigated.

Whether the EPO-R has the potential to activate the same signaling cascades in skeletal muscle (Figure 1) as in hematopoietic cells remains unclear. C_2_C_12_ myoblasts treated with EPO displayed an increase in JAK2, STAT5 (Ogilvie et al., 2000) and Akt phosphorylation (Jia et al., 2012); similar to signaling responses observed in neural cells. Furthermore, supraphysiological EPO concentrations activated Akt in mouse muscle (Hojman et al., 2009). However, STAT5 activation was not detected in rat skeletal muscle tissue following EPO stimulation (Lebaron et al., 2007). Acute EPO administration did not lead to any change in the phosphorylation levels of members of the STAT5, Akt and MAPK signaling pathways in human skeletal muscle (Christensen et al., 2012a). It is of interest to note that no EPO-induced response could be observed in endothelial and other non-hematopoietic cells, which had previously been described to be EPO-responsive (Sinclair et al., 2010). However, it must be noted that activation of Akt phosphorylation was also observed in the vehicle treated group, making it impossible to establish the specific effect of EPO on Akt signaling.

## Controversy surrounding EPO-R antibodies

Discrepancies between results obtained for EPO-R gene and protein expression or across species may be attributable to the non-specificity of certain commercially available EPO-R antibodies (Table 1).

**Table 1:** **Detectability of the EPO-R in skeletal muscle**.

**Study**	**Specie**	**Tissue**	**EPO-R gene expression (method)**	**EPO-R protein expression (method)**	**EPO-R antibody**
Ogilvie et al., 2000	Mouse	C2C12 myoblasts	Yes (northern blot)	Yes (WB)	Rabbit polyclonal antibody (Santa Cruz Biotechnology)
				Yes (immunohistochemistry)	
		Primary satellite cells	Yes (RT-PCR)	Yes (WB)	
				Yes (immunohistochemistry)	
		Embryos developing muscle	Yes (*in situ*-hybridization)	N/A	
Jia et al., 2009	Mouse	Primary myoblasts	N/A	Yes (immunohistochemistry)	M-20 clone (Santa Cruz Biotechnology)
Wang et al., 2012	Mouse	C2C12 myoblasts	Yes (RT-PCR)	Yes (WB)	EPO-R antibody (Santa Cruz Biotechnology)
Liu et al., 1994, 1997	Mouse	Muscle	No (RT-PCR)	N/A	N/A
Launay et al., 2010	Rat	L6 myoblasts	Yes (RT-PCR)	Yes (WB)	M-20 clone (Santa Cruz Biotechnology)
	Human	Primary myoblasts	Yes (RT-PCR)	Yes (WB)	
Rotter et al., 2008	Rat	Primary myoblasts	Transient (RT-PCR)	Transient (immunohistochemistry, data not shown)	unknown
Lundby et al., 2008a	Human	Muscle	Yes (RT-PCR)	Yes (immunohistochemistry)	MAB307 (R&D Systems)
Rundqvist et al., 2009	Human	Muscle	Yes (RT-PCR)	Yes (immunohistochemistry)	murine anti-human EPOR antibody (R&D Systems)
				Yes (WB)	C-20 clone (Santa Cruz Biotechnology) and MAB287 (R&D Systems)
		Isolated muscle fibers	Yes (RT-PCR)	N/A	
		Primary satellite cells	Yes (RT-PCR)	N/A	
Christensen et al., 2012a	Human	Muscle	N/A	Yes (WB)	M-20 clone (Santa Cruz Biotechnology)

A study comparing 4 commercially available antibodies showed that only the M-20 clone (sc-697, Santa Cruz Biotechnology) was suitable for detection of the EPO-R protein by Western Blot and that none, including the M-20 clone, was suitable for immunohistochemistry. The predicted molecular weight of the mature, membrane-bound form of the EPO-R protein is 56–57 kDa (Kuramochi et al., 1990; Nakamura et al., 1992; Fujita et al., 1997). While each antibody detected more than one band between 50–75 kDa, sequence analyses confirmed the EPO-R sequence in the 59 kDa band recognized by the M-20 antibody only (Elliott et al., 2006). Out of 6 commercially available antibodies, 5 of these antibodies detected rat or human EPO-R when overexpressed in HEK293 cells, but only 2 of these detected endogenous EPO-R in the human hematopoietic UT-7 cell line. Rodent brain is known to express high levels of the EPO-R (Yu et al., 2002); however, none of these antibodies was able to detect the EPO-R protein in rat brain homogenates. No certainty could be drawn either regarding the use of these various antibodies in immunohistochemistry (Kirkeby et al., 2007). These conflicting results illustrate the issues associated with various EPO-R antibodies. The EPO-R protein has been shown to be highly expressed in endothelial cells and other non-hematopoietic cells (Tramontano et al., 2003; Arcasoy, 2008; Noguchi et al., 2008). However, Sinclair et al. questioned the detectability of a functional EPO-R protein in non-hematopoietic cells. Using a previously validated, custom-made EPO-R antibody (Elliott et al., 2010), they demonstrated that the expression of the EPO-R protein was absent or 300- to 1000-fold lower in different types of endothelial cells when compared to hematopoietic UT-7 cells (Sinclair et al., 2010). However, UT-7 cells are engineered to translate 7 to 8 copies of the EPO-R gene (Chretien et al., 1994), resulting in a high expression level of the EPO-R protein (Kirkeby et al., 2007). Therefore, comparisons with physiological expression levels must be interpreted cautiously.

This controversy is well exemplified in human skeletal muscle. While the anti-EPO-R antibody used for immunohistochemistry by Rundqvist et al. was not precisely described (Rundqvist et al., 2009), the specificity of the antibody used by Lundby et al. (MAB307, R&D Systems) (Lundby et al., 2008a) has been questioned (Kirkeby et al., 2007). Indeed, the MAB307 clone exhibited a diffuse, non-specific staining in the cytoplasm, as well as some degree of nuclear staining in rat brain tissue, indicating non-specific binding. In muscle homogenates, Christensen et al. identified a band corresponding to the 59 kDa EPO-R seen in the positive control sample (K562 cell extracts) with the M-20 clone (Santa Cruz biotechnology), but not with the C-20 clone (Santa Cruz biotechnology), while Rundqvist et al. used the C-20 clone with the same positive control (K562 cell extracts) to assess the presence of the EPO-R in muscle (Rundqvist et al., 2009; Christensen et al., 2012a).

There are considerable concerns about the lack of specificity of numerous commercially available EPO-R antibodies. A more rigorous approach to measuring EPO-R protein levels in whole muscle homogenates and muscle cross-sections is therefore required. This should include the demonstration of appropriately validated EPO-R antibodies that detect not only an overexpressed exogenous EPO-R protein, but also detect in parallel the endogenous skeletal muscle EPO-R protein. The inclusion of negative control samples from muscle tissues or muscle cells that have an ablation of the EPO-R protein would be desirable. These positive and negative controls should be species specific for human and murine EPO-R proteins and include information relating to protein sequencing of the detected EPO-R protein bands.

## Effects of EPO in skeletal muscle

### In vitro

**Figure 2:**
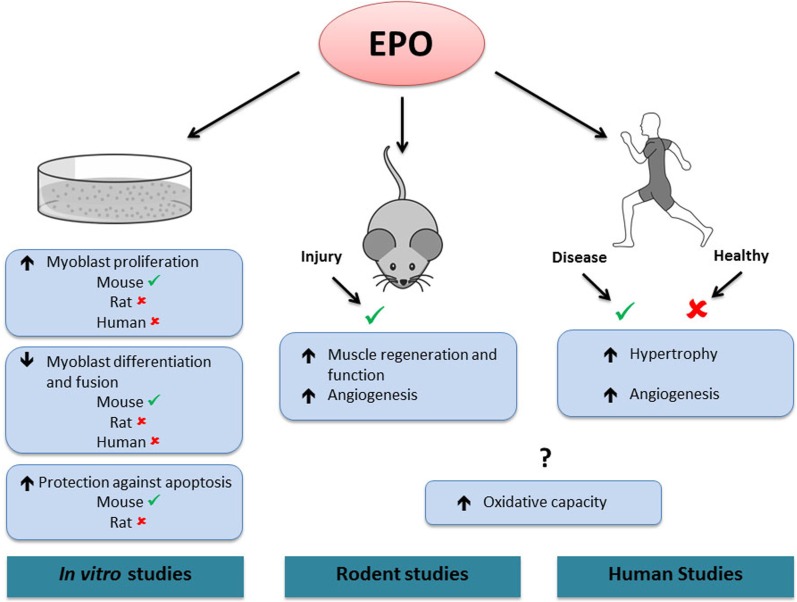
**Effects of EPO in skeletal muscle**.✓, activated by EPO. ✘, not activated by EPO. ?, contradictory results. *In vitro*, EPO treatment increases mouse, but not rat or human myoblast proliferation. EPO treatment decreases differentiation and fusion of mouse, but not rat or human myoblasts. EPO treatment protects against apoptosis in mouse but not in rat myoblasts. In rodents, EPO treatment increases muscle regeneration and angiogenesis following injury. In humans, EPO treatment increases skeletal muscle hypertrophy and angiogenesis in diseased conditions (chronic renal failure and Friedreich ataxia, respectively), but has no effect in healthy muscle. In both rodent and human studies, EPO has been shown to increase or have no effect on muscle oxidative capacity. Note that it is presently unknown if the effects of EPO treatment observed in rodent and human skeletal muscle are direct or indirect.

In line with its role in erythroid progenitor cells, EPO has been proposed to promote proliferation and survival in myoblasts (Figure 2). EPO stimulation enhanced proliferation and reduced differentiation and fusion of both satellite and C_2_C_12_ cells (Ogilvie et al., 2000; Jia et al., 2009). These effects were associated with an increase in the early myogenic regulatory factors (MRFs) Myf-5 and MyoD gene expression and a delay or inhibition in myogenin gene expression (Ogilvie et al., 2000). It was recently demonstrated that EPO exerted its positive effects on myoblast proliferation and regulated MRFs via its induction of sirtuin1 (Sirt1), GATA-4 and T-cell acute leukemia 1 (TAL1) (Wang et al., 2012). Similarly, MRFs displayed an expression pattern characteristic of the early differentiation stages in C_2_C_12_ cells overexpressing the EPO-R cultured in differentiation media (Jia et al., 2009). The same study showed that EPO treatment protected myoblasts against hypoxia-induced apoptosis. It was also proposed that EPO supplementation alone (Rotter et al., 2008) or coupled with enhanced EPO-R expression (Jia et al., 2009) promoted rat and mouse myoblast survival, respectively, under serum-starved conditions. Mouse myoblasts extracted from muscle lacking the EPO-R did not proliferate in culture, while a higher proliferation and survival rate was observed in myoblasts extracted from transgenic mice with chronically elevated circulating EPO (Jia et al., 2012). In contrast to these results, EPO treatment did not promote proliferation or postpone differentiation in rat or human myoblasts cultured in either normoxic or hypoxic conditions (Launay et al., 2010). Furthermore, EPO supplementation did not decrease apoptosis in rat myoblasts (Rotter et al., 2008). While several types of recombinant EPO molecules have been used in these studies (epoetin-alpha and/or -beta), the doses used for *in vitro* treatments were reasonably consistent between studies (generally 0.5–10 IU/mL), except for the study by Rotter et al. who used 50 IU/mL.

### In rodent models

Expression of the EPO-R in non-hematopoietic cell lineages is not necessary for normal mouse development. Transgenic mice expressing the EPO-R exclusively in the hematopoietic lineage displayed normal development, fertility and survival (Suzuki et al., 2002). However, the lack of EPO-R in non-hematopoietic tissues seems to compromise muscle regeneration and repair following injury. In a model of cardiotoxin-induced muscle injury, mice with EPO-R expression restricted to hematopoietic tissues had a reduced number of satellite cells and were more sensitive to muscle injury than wild-type controls (Jia et al., 2012).

It has been suggested that EPO exposure could improve muscle function in trauma or disease conditions. Local EPO treatment increases rat soleus muscle regeneration and strength following mechanically induced injury (Figure 2); a response associated with increased proliferating satellite cells numbers (Rotter et al., 2008). Systemic EPO treatment has a similar effect following a combined muscle-nerve injury. Histological analysis of muscle tissue demonstrated increased proliferation of satellite cells and reduced cell apoptosis (Rotter et al., 2012). Mice with chronically elevated circulating EPO displayed reduced injury and accelerated recovery (Jia et al., 2012). In a model of severe mouse sepsis, EPO treatment rapidly increased capillary density and led to a normalization of skeletal muscle microcirculation and tissue metabolism (Kao et al., 2007). Interestingly, C_2_C_12_ myoblasts overexpressing the EPO-R transplanted into muscle fibers were able to restore dystrophin protein expression in *mdx* mice, a mouse model for Duchenne muscular dystrophy (Jia et al., 2009). Furthermore, this effect was enhanced by EPO administration.

Several groups used EPO deficient mouse models to investigate the potential of a direct or indirect role of EPO in skeletal muscle development and function. No difference in muscle fiber distribution, fatigability or tetanic force was reported for EDL and soleus muscle of EPO deficient mice, either in normoxic or in hypoxic conditions, when compared to wild-type controls (Hagstrom et al., 2010b). EPO deficiency did not result in muscle fiber atrophy or microvessel network alteration, two markers of a poor EPO function in human (Hagstrom et al., 2010a). However, microarray analysis in skeletal muscle from EPO-deficient mice identified increases in genes related to muscle hypoxia, proteolysis, cell death and apoptosis as well as reductions in genes involved in glycolysis and mitochondrial function. These findings support the hypothesis that EPO has a protective effect in muscle (Mille-Hamard et al., 2012). Although there is conflicting evidence, some data support the role of EPO in a protection against disease or trauma. However, its presence may not be required for the maintenance of normal adult muscle function.

Several studies recently suggested that EPO treatment might affect the oxidative properties of skeletal muscle and induce a shift towards a more oxidative phenotype. In both sedentary and exercising rats, 2 weeks of EPO treatment increased the activity of several oxidative enzymes in different muscles, including cytochrome C oxidase in the soleus muscle but not in the vastus lateralis or in the gastrocnemius muscles. A general increase in the expression of slow twitch myosin light chains and oxidative myosin heavy chains following EPO treatment was also reported following endurance training. Combining training with EPO had additive effects (Cayla et al., 2008). In contrast to these results, rats receiving EPO displayed significant decreases in the expression of proteins involved in the mitochondrial biogenesis pathway, such as PGC-1α, mTFA and cytochrome c, in the gastrocnemius but not in the soleus muscle (Martinez-Bello et al., 2012). EPO was also suggested to prevent the impairment of mitochondrial structure and function induced by a local anaesthetic in rat muscle (Nouette-Gaulain et al., 2009). Alternatively to EPO administration, EPO gene electrotransfer results in supraphysiological circulating levels of EPO in muscle. In this model, when placed on a high fat diet, mice gained less body fat and presented muscle hypertrophy as well as increases in oxidative myosin heavy chain I mRNA expression, vascularization, lipid oxidation and expression of genes involved in fat metabolism and thermogenesis (Hojman et al., 2009). However, these mice displayed a 100-fold higher serum EPO concentration when compared to WT; therefore, the physiological relevance of such results is questionable. On the other hand, it has previously been shown that hyperoxia could augment muscle oxidative capacity (Ploutz-Snyder et al., 1996), although other studies have failed to reproduce similar results (Perry et al., 2007; Layec et al., 2012). It can therefore be hypothesized that an EPO-induced increase in oxygen supply could mediate a shift towards a more oxidative muscle, especially at supraphysiological EPO concentrations. Furthermore, this adaptive process may be completely independent of the EPO-R.

These inconsistent and inconclusive results obtain from *in vitro* and *in vivo* rodent studies question the role of EPO in muscle cell differentiation and survival and highlight the need for more standardized studies. Ideally, mouse models displaying skeletal muscle specific EPO-R knockout or over expression would be an important tool to assess the direct importance and role of EPO and EPO-R in skeletal muscle. The use of a Cre/loxP system to specifically knockout a target of interest in mouse skeletal muscle *in vivo* has been developed (McCarthy et al., 2012). This inducible model is able to overcome limitations of traditional gene targeting strategies and can down regulate the target of interest in a temporal and tissue-specific manner in the adult animal following tamoxifen administration. For over expression studies in adult animals, the use of adeno-associated viruses (AAV) has now become a popular method to deliver stable gene targets of interest into skeletal via intramuscular injection (Joanne et al., 2013) or systemically via intravenous (Katwal et al., 2013) or intraperitoneal administration (Charan et al., 2012).

### In humans

In human skeletal muscle *in vivo*, a single EPO injection significantly increased the mRNA content of the myogenic regulatory factor MRF-4 within 10 h (Lundby et al., 2008b). As resistance exercise also increases MRF-4 transcription, it was suggested that EPO might play a role in muscle fiber growth (Psilander et al., 2003). Sixty-five minutes of cycling exercise resulted in an increase in EPO-R mRNA levels in muscle biopsies collected 2 h post-exercise when compared to pre-exercise levels (Rundqvist et al., 2009). Recently it was shown that an acute systemic EPO injection did not increase the phosphorylation of members of STAT5, MAPK, Akt and NFKB pathways in skeletal muscle (Figure 2) when measured between 1 and 6 h following the EPO injection (Christensen et al., 2012a). In addition, EPO-R phosphorylation remained unchanged following EPO administration, although the use of a phospho-EPO-R antibody in muscle extracts has very little precedent in the literature. EPO administration also increased resting energy expenditure but not insulin sensitivity. While the mechanism controlling the EPO-induced increase in energy expenditure was not established, there were no changes in skeletal muscle mRNA expression of UCP-2, UCP-3, CPT-1 or PPAR-α or in AMPK or ACC phosphorylation (Christensen et al., 2012b). Fourteen weeks of EPO treatment had no effect on either muscle fiber hypertrophy or angiogenesis (Lundby et al., 2008b). Interestingly, it has been reported that patients with chronic renal failure treated with EPO showed an increase in muscle fiber diameter, especially in type I fibers, as well as an increase in glycogen content when compared to non-treated patients (Davenport et al., 1993). Long term EPO treatment had no effect on the muscle membrane transport system, on the enzymes involved in pH regulation or on the majority of proteins involved in acid–base balance. It had no effect either on cytochrome C or on the glycolysis enzyme hexokinase (Juel et al., 2007), a result in accordance with those of Martinez-Bello (Martinez-Bello et al., 2012) in the mouse soleus but not gastrocnemius muscle. However, in line with previous results in rodents (Cayla et al., 2008; Hojman et al., 2009), 8 weeks of EPO treatment enhanced skeletal muscle mitochondrial oxidative phosphorylation and maximal electron transport capacity (Plenge et al., 2012). Interestingly, prolonged EPO administration increased muscle capillary density in Friedreich Ataxia patients and may therefore contribute to the improved motor function reported following EPO treatment (Nachbauer et al., 2012). Altogether, these findings suggest that in skeletal muscle *in vivo*, EPO may not activate the EPO-R or the established EPO signaling cascades, such as JAK2/STAT5 or PI3-K/Akt. Additionally, the heterodimer receptor combining the EPO-R and the β cR subunit, which was proposed to partially or completely mediate the non-hematopoietic functions of EPO, could not be detected in skeletal muscle at the gene or protein level (Rundqvist et al., 2009). Therefore, the effects of EPO administration on human skeletal muscle growth and repair or metabolism may not be exerted by a direct EPO/EPO-R interaction, but rather be the consequence of a better oxygen supply to the tissues.

## Conclusion

The potential roles of EPO and its receptor in skeletal muscle have raised frequent speculation, partially due to its widespread use in endurance sport. Indeed, it has been suggested that EPO could have a positive effect on skeletal muscle regeneration, growth, oxidative capacity and angiogenesis. However, the role of EPO in skeletal muscle remains unclear and it is yet to be determined whether its effects on the skeletal muscle lineage are comparable to what is seen with the hematopoietic lineage. This review provides a comprehensive picture of the work completed in this area and highlights the strong need for more stringent, rigorous and consistent studies in the future. We suggest that researchers consider the different *in vitro* responses of immortalized cell lines such as mouse C2C12 and rat L6 muscle cells when compared with primary muscle cell lines, as well as species differences between human and rodent primary muscle cell lines. The use of overexpression or knockdown strategies that target the EPO-R specifically in the adult skeletal muscle will significantly advance our understanding of the role that EPO and the EPO-R play in skeletal muscle biology. Finally, researchers should be aware of the numerous commercially available EPO-R antibodies that are non-specific and be able to demonstrate unequivocally the specificity of their chosen EPO-R antibody to detect the specific EPO-R protein.

### Conflict of interest statement

The authors declare that the research was conducted in the absence of any commercial or financial relationships that could be construed as a potential conflict of interest.
